# Medical Topography, Epidemics of the United States, Etc.

**Published:** 1855-09

**Authors:** 


### MEDICAL TOPOGRAPHY, EPIDEMICS OF THE UNITED STATES, ETC.—COMMITTEE OF THE AMERICAN MEDICAL ASSOCIATION.

At the last annual meeting of the American Medical Association in Philadelphia, May, 1855, a committee was appointed, of one member from each State and Territory, and one from the Army and one from the Navy of the United States, to report upon the medical topography and the epidemic diseases of the United States, and the most successful treatment of the latter.

The following form of a circular has been agreed upon:

CIRCULAR.

The Committee of the American Medical Association "on Medical Topography, Epidemic Diseases, and most successful treatment thereof," address you this circular in their endeavor to get together materials for a medical history of the country. Please communicate to the address of the undersigned any and all information which may enable him to make a report, in which due credit will be given to each collaborator, and his name mentioned in connection with facts and histories furnished by him.

Please mention everything that has been printed or published about the medical history of your district, and topographical accounts of histories of particular epidemics, and say how far your own observations enables you to vouch for facts therein presented.

Geological and physical charts are very desirable, as well as descriptions of peculiar features of country or city.

Please mention *all epidemics* of which you may have any knowledge, being particular to assign limits of time and space as exactly as possible, giving, in connection with each disease, the peculiar features of the country, city, ward or street where it prevailed, with slope of rocks, character of soil, meteorological records and observations, altitude above the ocean or adjacent bodies of water, character of the water, artificial changes, as by cultivation, cutting down or planting of trees, sewerage, drainage, etc., etc.

Any supposed causes of disease, peculiar symptoms, post mortem appearances, prevention, therapeutical influences, and all details of age, sex, nativity, occupation, etc., of individuals, and of the duration and severity of disease at different periods, proportion of mortality, etc., should be given.

An early answer to this communication is desired.

[Those in Kentucky will please address Dr. W. L. Sutton, Georgetown, Ky.]

